# Fluorescent amplification for next generation sequencing (FA-NGS) library preparation

**DOI:** 10.1186/s12864-020-6481-8

**Published:** 2020-01-28

**Authors:** Jennifer Chiniquy, Megan E. Garber, Aindrila Mukhopadhyay, Nathan J. Hillson

**Affiliations:** 1 Biological Systems & Engineering Division, Lawrence Berkeley National Laboratory, Berkeley, CA 94720 USA; 2 U.S. Department of Energy Agile BioFoundry, Emeryville, CA USA; 3 Department of Comparative Biochemistry, University of California, Berkeley, CA 94720 USA

**Keywords:** Library preparation, Next generation sequencing, NGS, SYBR green, Echo, High-throughput, qPCR

## Abstract

**Background:**

Next generation sequencing (NGS) has become a universal practice in modern molecular biology. As the throughput of sequencing experiments increases, the preparation of conventional multiplexed libraries becomes more labor intensive. Conventional library preparation typically requires quality control (QC) testing for individual libraries such as amplification success evaluation and quantification, none of which occur until the end of the library preparation process.

**Results:**

In this study, we address the need for a more streamlined high-throughput NGS workflow by tethering real-time quantitative PCR (qPCR) to conventional workflows to save time and implement single tube and single reagent QC. We modified two distinct library preparation workflows by replacing PCR and quantification with qPCR using SYBR Green I. qPCR enabled individual library quantification for pooling in a single tube without the need for additional reagents. Additionally, a melting curve analysis was implemented as an intermediate QC test to confirm successful amplification. Sequencing analysis showed comparable percent reads for each indexed library, demonstrating that pooling calculations based on qPCR allow for an even representation of sequencing reads. To aid the modified workflow, a software toolkit was developed and used to generate pooling instructions and analyze qPCR and melting curve data.

**Conclusions:**

We successfully applied fluorescent amplification for next generation sequencing (FA-NGS) library preparation to both plasmids and bacterial genomes. As a result of using qPCR for quantification and proceeding directly to library pooling, the modified library preparation workflow has fewer overall steps. Therefore, we speculate that the FA-NGS workflow has less risk of user error. The melting curve analysis provides the necessary QC test to identify and troubleshoot library failures prior to sequencing. While this study demonstrates the value of FA-NGS for plasmid or gDNA libraries, we speculate that its versatility could lead to successful application across other library types.

## Background

Next generation sequencing (NGS) is becoming a predominant tool in answering a broad range of biological questions. Its popularity can be attributed to its cost-effectiveness, its broad utility, and its multiplexing capabilities, which can be used to sequence hundreds, if not thousands, of individual libraries simultaneously [1]. Because library preparation is step-intensive and cumbersome, multiplexing comes with a series of technical challenges [2]. Most notably, after DNA purification, each individual library requires individual quality controls (QC) to determine if the library amplified and the concentration after amplification. The concentration of each library is then used to determine the relative quantities of DNA so that they can be pooled in equal amounts. Accurate quantification of NGS libraries is important to ensure evenness of library pooling before sequencing. Current strategies available for quantifying NGS libraries after amplification and purification include Quant-iT dsDNA Assay Kit (Thermo Fisher Scientific, Waltham, MA), real-time qPCR-based library quantification methods like the KAPA Library Quantification Kit (Roche, Pleasanton, CA) [3], and digital PCR [4–6]. Each of the aforementioned quantification methods have been validated for NGS library preparation, but are notably laborious when applying them to high-throughput workflows. Modifications to the standard library preparation workflow must be considered to make multiplexing a more viable option for researchers. Our goal was to establish a modified library preparation workflow that eases multiplexing by limiting the number of steps required per individual library. We accomplished this by bridging amplification and QC steps to bypass individual DNA purification using real-time quantitative PCR (qPCR). We call this library preparation workflow modification fluorescent amplification for NGS (FA-NGS).

A wide range of applications rely on qPCR, including genotyping analysis, medical diagnostics, gene expression profiling [7], phytopathogen identification [8], forensic studies [9], and validation of DNA microarrays [10]. In the context of NGS, qPCR has been used primarily for the quantification of NGS libraries, which is used to pool purified libraries in equal amounts, a crucial step in preventing poor quality sequencing data [11]. As opposed to employing quantification after DNA amplification and purification of individual libraries, we hypothesized that we could replace these two steps with a single qPCR step using SYBR Green I.

In addition to reducing the number of steps in the NGS library preparation workflow, qPCR with SYBR green I added supplementary benefits to the modified NGS workflow. qPCR is a widely used technique for nucleic acid detection and quantification that employs polymerases together with intercalating fluorescent dyes or optionally fluorescently labeled sequence-specific probes. The development of inhibitor-tolerant fusion polymerases such as the Pfu-Sso7d polymerase [12, 13], which is used with SYBR green I allows for flexibility in reaction conditions, including nucleic acid sample input types with NGS library preparation reagents. Other advantages include a wide dynamic range (up to 8 logs), and high sensitivity even with low volumes or low input quantities. Finally, because the data is collected in a closed-tube system, there is reduced risk for sample contamination [14].

A limitation of using PCR dyes such as SYBR is a lack of binding specificity. These nonspecific dyes, unlike probe-based assays, intercalate with any dsDNA including primer-dimer, which can lead to false positives. However, since the melting temperature of primer dimer is typically much lower than the amplicons of interest, melting curve analysis (MCA) at the end of qPCR enables easy detection of amplicon over primer dimers. We hypothesized that we could use MCA, enabled by qPCR, to determine if individual libraries were properly amplified without any additional reagents or costs.

To evaluate whether a combination of qPCR and MCA could be applied to NGS library construction, we modified two distinct library workflows. We first tested FA-NGS with Illumina’s Nextera XT (Illumina, San Diego, CA). The standard workflow uses enzymatic fragmentation with transposases followed by conventional PCR amplification with indexed primers, purification, and quantification. Our workflow modifications included replacing PCR and the final quantification with low volume qPCR and SYBR Green I, and adding MCA. We also adopted additional modifications for the Nextera workflow, which have been previously shown to have utility for multiplexing NGS libraries such as automation using Echo acoustic liquid handling and reduction in reaction volumes [15–17]. We next evaluated FA-NGS with adapter ligation-based library construction kit using the NEBNext Ultra II DNA library preparation kit. The standard workflow includes DNA shearing, a multi-step process for adapter ligation, intermediate purification and quantification, and PCR with indexed primers followed by a final purification and quantification. As with the first NGS library workflow, our modifications included replacing PCR and the final quantification with low volume qPCR and SYBR Green I, and adding MCA.

**Fig. 1:**
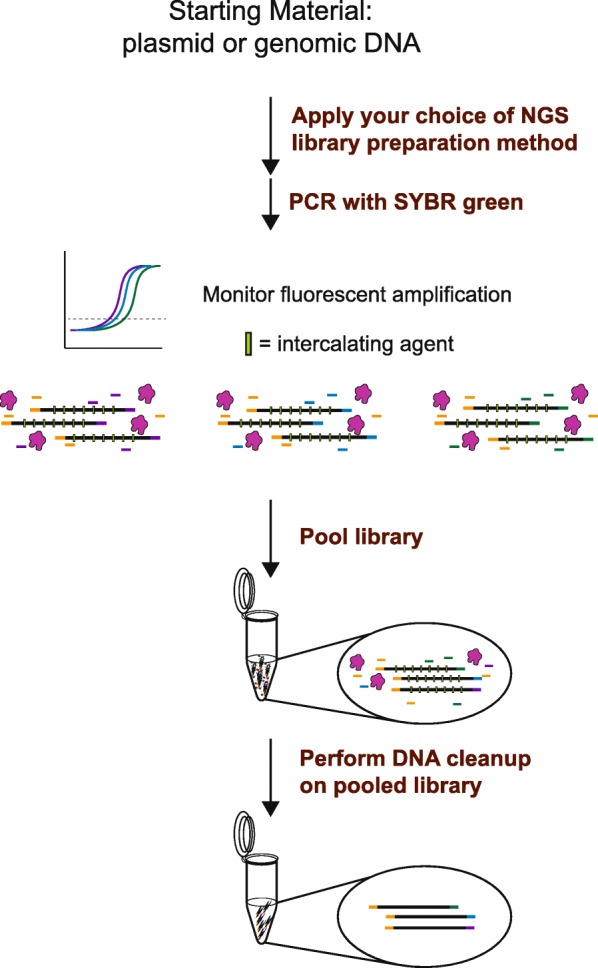
*FA-NGS Workflow*: Following library preparation method of choice, amplification is applied with SYBR green mastermix, including polymerase (pink clouds), intercalating dye (green rectangles), and index primers (yellow, blue, purple rectangles). The fluorescence is monitored during library amplification. Melting curve analysis is then applied to an aliquot of the library to determine amplification success. If libraries pass the melting curve analysis QC, end RFU measurements are used to pool the amplified libraries in equal quantities. Libraries that fail melting curve analysis QC are omitted from subsequent steps. The pooled library is then purified and ready for NGS

The libraries from both modified workflows utilizing FA-NGS (Fig. 1, Additional file 1: Fig. S1) were sequenced in this study. These modifications reduced the hands-on time to construct the library, the PCR reaction volume, and the overall risk of sample contamination [14]. We found that by replacing conventional PCR with qPCR in NGS library preparation, we were able to successfully use quantified measurements of fluorescence as a proxy for relative concentration to pool 96 individual libraries (Fig. 1, Additional file 1: Fig. S1). Because we replaced PCR with qPCR, we were also able to perform MCA as an intermediate QC to confirm library amplification. This was done with a simple procedure and did not incur additional reagent cost.

## Results

**Fig. 2:**
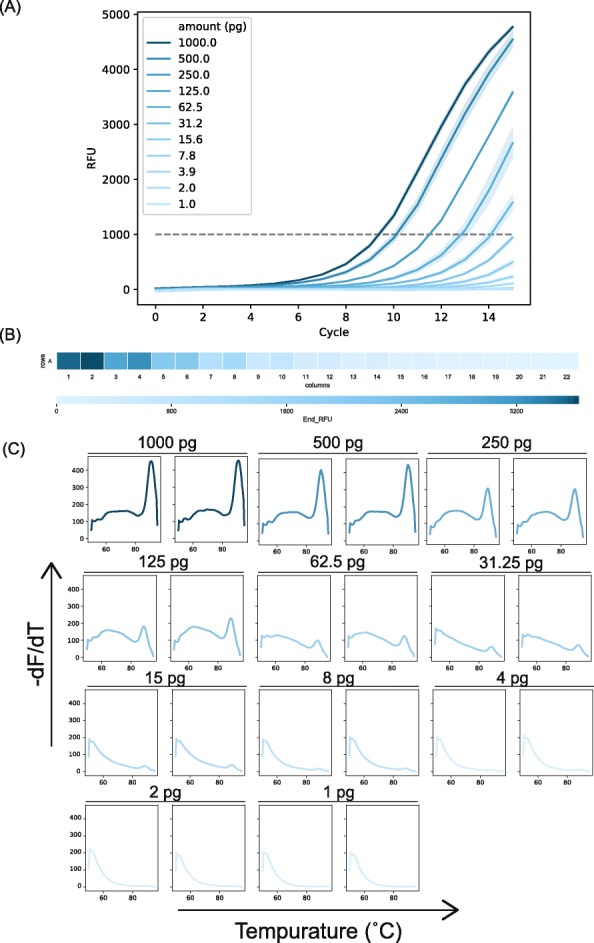
*Input titration of AL-DNA monitored with FA-NGS:* DNA diluted 2-fold starting at 1000 pg per reaction was amplified with SYBR green in duplicate and monitored with continuous fluorescence (**a**), end-fluorescence (**b**), and melting curve analysis (**c**)

We chose to apply FA-NGS to two commonly used methods for preparing libraries. We used Nextera-based library preparations of plasmid DNA and adapter ligation-based library preparations of purified genomic DNA (gDNA). Using an input titration assay, we determined that the concentration of input adapter-ligated (AL) gDNA or plasmid could be tracked by FA-NGS with NGS primers (Fig. 2, Additional file 2: Fig. S2 and Additional file 3: Fig. S3). We observed that we could apply Nextera tagmentation and FA-NGS to plasmids with starting amounts as low as 1.5 pg per 7.5 μL reaction. We also found that the detection limit of FA-NGS above a 1000 end relative fluorescence unit (RFU) threshold for AL libraries was about 62.5 pg per 7.5 μL reaction (Fig. 2a). For gDNA samples, the end RFU values scaled with the 2-fold dilution factor of the starting amount of the input (Fig. 2b). This indicated that the end RFU values could be used to estimate the transfer volumes required to generate a library of approximately equal concentrations of each indexed sample. MCA of the AL gDNA input showed that even samples with input below 62.5 pg still have amplified DNA, as observed by a high melting temperature peak (Fig. 2c, Additional file 4: Fig. S4B). For input values below 8 pg, no such high temperature peak was observed.

**Fig. 3:**
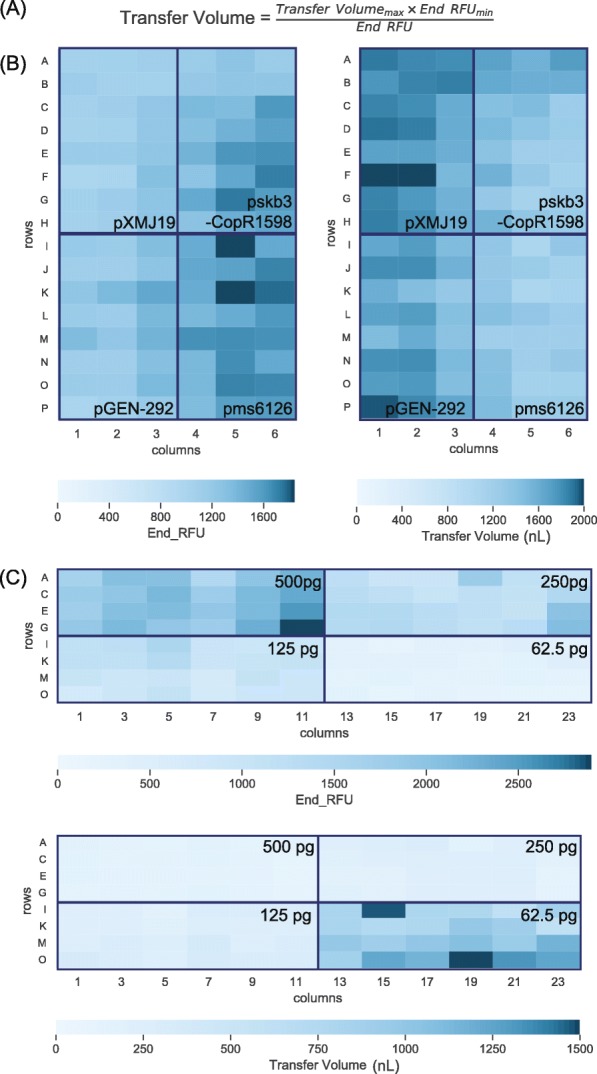
*End RFU and transfer volumes of Nextera and AL library preparations:* Equation for calculating transfer volumes from End RFU values, where Transfer Volume_max_ is determined by the user, and End RFU_min_ is the minimum End RFU value in the data set (**a**). Heatmaps of end RFU values and the corresponding calculated transfer volume of each well, 4 unique plasmids per quadrant of Nextera library prepared plasmids (**b**), 4 two-fold dilutions starting at 500 pg per quadrant of AL library prepared gDNA (**c**)

Combinations of dual index primers were used to uniquely index 96 samples. The plasmid library consisted of 4 plasmids (Table 1) with 24 replicates for each. The gDNA library was prepared with gDNA isolated from the same organism, *Pseudomonas stutzeri* RCH2, with 24 replicates of 4 starting amounts. The libraries were amplified and measured with continuous fluorescence. End RFU values were used as a proxy for relative concentration of each individual library. As expected, wells with similar starting amounts yielded similar end RFU values (Fig. 3b, Additional file 4: Fig. S4).

**Table 1:** *Plasmids used in Nextera library preparation.* All plasmids used are available through the public instance of the ABF registry [18]. See the availability of data and materials section for additional information

Plasmid	Resistance	Size	Reference	Registry ID
pXMJ19	Chloramphenicol	6592	[19]	ABFPUB_000064
pGEN-292	Kanamycin	10,370	(Kirby et al. unpublished)	ABFPUB_000068
pskb3-CopR1598	Kanamycin	6029	[20]	ABFPUB_000072
pms6126	Carbenicillin	4038	[21]	ABFPUB_000070

MCA (Additional file 5: Fig. S5 and Additional file 6: Fig. S6) was applied to assess the overall quality of each indexed sample. This enabled us to predict individual library failure prior to sequencing. As expected, a late melting peak was observed for wells with the appropriate template DNA. Samples without DNA template or with degraded DNA template were expected to have a low temperature peak, an indication of primer dimer and lack of library amplification (Fig. 2c, Additional file 3: Fig. S3).

**Fig. 4:**
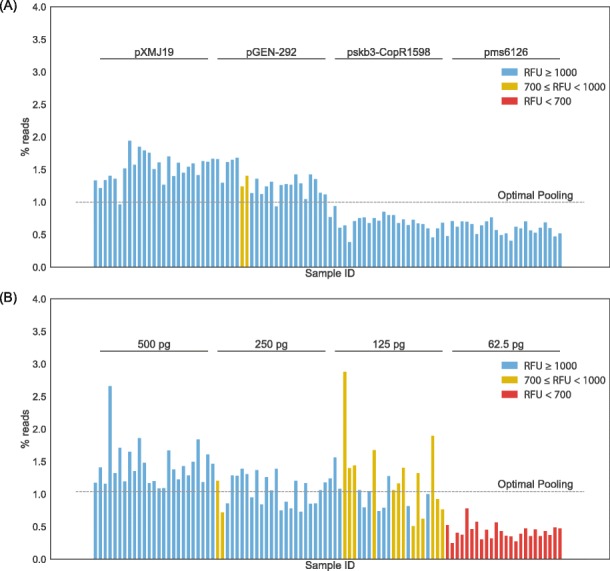
*Library pooling yields near even distribution of percent of the total reads:* Optimal pooling is the expectation (1.04) of the percent reads if all 96 libraries pooled evenly. 4 unique plasmids of Nextera library prepared plasmids (**a**), 4 two-fold dilutions starting at 500 pg of AL library prepared gDNA (**b**). The bars are colored by the end RFU values of the qPCR, blue if the RFU was greater than 1000, yellow if the RFU was between 700 and 1000, and red if the RFU was less than 700. For gDNA library (**b**), samples below RFU 700 were likely to be underpooled because they were out of the linear range of the qPCR. This trend was not observed with the plasmid library (**a**)

To determine whether the end RFU values were a reasonable measurement of the final concentration of DNA after amplification, we sequenced the two libraries consisting of 96 combinations of dual indices with the Illumina MiSeq Reagent Nano kit. We observed that the percent of reads of each sample was comparable in both Nextera and AL libraries (Fig. 4). Both the Nextera and AL libraries yielded similar distribution of percent reads with a *p*-value of 1 (Additional file 7: Fig. S7). While each sample is not pooled at exactly 1.04% of the reads (expectation for optimal pooling from 96 samples), a majority of the samples from each library do fall below a 50% difference from optimal pooling range (Additional file 8: Fig. S8). Sequencing quality value scores for the PhiX Control Library and for the FA-NGS libraries were above the specification provided by Illumina of at least 80% Q30 [22] (Additional file 9: Fig. S9).

## Discussion

In this report, we provide the validation of time-saving modifications to two common workflows for NGS library preparation. By implementing FA-NGS, we show that we can monitor amplification of each sample within an NGS library, perform quantification during qPCR to determine library normalization ratios for sequencing, and simultaneously perform a simple QC to confirm the amplified DNA template is present.

FA-NGS allows for accurate pooling directly after PCR, reducing the risk of user error. Typical NGS library preparation requires individual library purification prior to QC and pooling (Additional file 1: Fig. S1), which could lead to sample mix up or loss of library. Alternatively, unpurified and unquantified libraries could be pooled in equivolume amounts, however there would be no indication that the library amplified (increasing the potential for pooling primer dimer which would have deleterious effects during sequencing [23]) or that the samples varied in concentration. To avoid the latter issue, many PCR cycles (> 20 cycles) could be applied to fully saturate the reactions, however, this introduces the caveat of increased PCR bias, which can impact downstream interpretation of sequencing results. FA-NGS resolves these problems with a single step of amplification and quantification.

MCA enables users to perform individual library QC testing without DNA purification. Many workflows, especially Nextera-based library preparations lack QC tests until the final step of the protocol. When used on small aliquots of the amplified libraries, MCA confirms if an amplified library product is present. In applying FA-NGS, we suggest removing wells containing samples without amplified library, as determined by MCA, from the pooling calculations. Samples without amplified library may contain primer dimer, which if not removed in a final size selection can impact sequencing run quality and cause over clustering since smaller DNA fragments cluster more efficiently [24]. In accordance with our goal to make a user-friendly NGS library preparation workflow for multiplexed libraries, MCA adds an important QC step without additional costs or reagents.

The modified NGS workflow strategies described here are most beneficial for high-throughput NGS library applications, due to their compatibility with automation systems such as the Labcyte Echo. In order to make this modified workflow accessible to researchers with high-throughput NGS workflows, we made a FA-NGS software tool available on GitHub (see availability of data and materials) that can be used for set-up, analysis, and pooling of FA-NGS libraries. The “set-up tool” outputs a. CSV file for primer transfer on the Echo acoustic liquid dispensing system. The “analysis tools” enable rapid determination of sample failure from end RFU and melting curve measurements. These tools output heatmaps of the end-fluorescent values and small multiple MCA plots in the plate layout of the user’s choice. The “pooling calculator” outputs a. CSV file, compatible with the Echo acoustic liquid dispensing system, for pooling libraries based on end RFU values.

A common goal in quantifying individual samples within a pooled library is to ensure that one given sample is not overrepresented as compared to another. Because of the potential variation in DNA shearing or fragmentation size distributions from NGS library preparation, it may be recommended to both measure the library concentrations and perform individual library size analysis to enable pooling in equimolar amounts. However, individual library size analysis for high-throughput sequencing applications can be laborious and costly. We demonstrate using relative fluorescence from qPCR to normalize library concentrations, without applying individual sizing analysis, that the distribution of sequencing reads (Fig. 4, Additional file 7: Fig. S7 and Additional file 8: Fig. S8) from using the FA-NGS library quantification strategy is sufficient to give comparable representation of 96 libraries.

To further validate the FA-NGS workflow, we performed sequencing quality assessment and included the Illumina PhiX Library Control for comparison. All libraries surpassed the recommended quality value score (Additional file 9: Fig. S9), per Illumina’s specification of the percentage of bases at or above Q30, indicating that the FA-NGS workflow successfully generated high quality sequencing reads.

While implementing FA-NGS has many benefits, the apparent pitfalls must be noted. We observed that samples in the gDNA library with end RFU values below 700 RFU were under-pooled (Figs. 3b and 4b). Because these samples were out of the fluorescence linear range of the qPCR, it is likely that the end-fluorescent values for these samples could not be used as a precise proxy for the relative DNA concentration and were therefore transferred at a volume that overestimated the relative concentration. We did not observe this trend with the plasmid library. We speculate that variation in percent reads for plasmid libraries could be an effect of amplifying beyond the exponential phase to the plateau phase of qPCR, such that the end-fluorescent values used for pooling were less precise (Additional file 10: Fig. S10). For standard library preparation workflows that use conventional PCR, there could be a comparable limitation during the final library quantification step, depending on the fluorescence linear range of the DNA quantification assay [25, 26]. For high-throughput applications employing FA-NGS, it may actually be preferable to allow amplification to the plateau phase, since initial sample concentrations or sample types may vary and the additional qPCR cycles help ensure all samples are sufficiently amplified.

The libraries in this report were sequence validated with Illumina Miseq Nano kits as proof of principle for our modified library workflow. Although the Nano kits give relatively low read counts compared to other Illumina sequencing kits, they generated more than sufficient coverage for the 96 plasmids (Additional file 11: Table S1). However, for deep sequencing such as for bacterial genomes, larger MiSeq, HiSeq, or NovaSeq kits are generally recommended.

In this study, our modified workflow was successfully applied towards sequencing of full plasmids and bacterial genomes. These streamlined library preparations improve QC testing capability while reducing the total number of steps required to generate libraries.

## Conclusions

Here we present a useful modification to conventional NGS library preparation workflows, FA-NGS, which was successfully incorporated into Illumina Nextera XT and NEBNext Ultra II DNA library preparation. We demonstrate the FA-NGS workflow ease of use with fewer overall steps than conventional library workflows, as well as an MCA QC test to confirm successful library construction before sequencing. An open source FA-NGS software tool is available to assist in implementing the workflow (see availability of data and materials). We expect that the application of FA-NGS will greatly benefit the production of any NGS library type which is amplified by PCR.

## Methods

### Plasmid DNA

Plasmid DNA was extracted from *E. coli* using the PureYield Plasmid Miniprep System (Promega, Madison, WI). *E. coli* cultures were grown overnight in LB medium supplemented with the appropriate resistance markers. DNA was quantified by the Qubit dsDNA HS Assay (Invitrogen, Carlsbad, CA). Each plasmid was diluted to 0.1 ng/μl for use in library construction. Plasmids are listed in Table 1. All plasmids used are available through the public instance of the ABF registry [18]. See the availability of data and materials section for additional information.

### Enzymatic fragmentation and adapter ligation of plasmids

Plasmids were fragmented and ligated to partial Illumina adapters sequences in a single step using the Nextera XT DNA Library Preparation Kit (Illumina, San Diego, CA). Reactions were set up as described previously [27]. Using the Labcyte Echo 550 acoustic liquid dispensing system (Labcyte, Sunnyvale, CA), the Nextera tagmentation reaction volume was reduced to 1 μL total. Samples were incubated at 55 °C for 5 min for fragmentation and ligation, then at 70 °C for 15 min for heat inactivation of transposase enzymes. This reaction was immediately followed by qPCR.

### Nextera library amplification of using real-time qPCR

Pairwise 8-nucleotide barcodes and the remainder of the Illumina adapter sequences were added using primers from the Nextera XT Index Kit v2. Primers were transferred using the Echo 550. Real-time qPCR and concurrent amplification and relative quantification of the library was facilitated on the CFX384 Touch Real-Time PCR Detection System using SsoAdvanced Universal SYBR Green Supermix (Bio-Rad, Hercules, CA). The Biomek Nx S8 was used to add SYBR Green and water. Reaction volumes were 7.5 μL total, with 0.25 μL each of the indexed Nextera primers, 3.75 μL of SYBR Green, 2.25 μL water, and 1 μL of the previous reaction. Cycling parameters were 72 °C for 3 min, 98 °C for 30 s, followed by 20 cycles of 10 s at 98 °C, 30 s at 63 °C and 3 min at 72 °C. The end RFU values were determined with CFX Manager software (Bio-Rad, Hercules, CA) for all libraries.

### Shearing and adapter-ligation of genomic DNA

*Pseudomonas stutzeri* RCH2 was grown overnight in 5 mL UGA media [20]. UGA media contained 4.7 mM ammonium chloride, 1.3 mM potassium chloride, 2 mM magnesium sulfate, 0.1 mM calcium chloride, 0.3 mM sodium chloride, 5 mM sodium dihydrogen phosphate, 20 mM sodium lactate, and 25 mM MOPS. Vitamins and minerals were added as described by Widdel and Bak [28]. Genomic DNA was extracted using the Wizard genomic kit (Promega, Madison, WI) per the manufacturer’s protocols. The resulting genomic DNA was diluted to 30 ng/μL and sheared with Covaris microTubes (Covaris, Woburn, MA) using the standard protocol for shearing DNA to 200 base pairs. Following shearing, 1 μg of sheared genomic DNA was ligated to adapters using the NEBNext Ultra II DNA library preparation kit (New England Biolabs, Ipswich, MA) according to the manufacturer’s instructions without size selection and up until the final PCR. Size distribution of the adapter ligated DNA was verified on the Bioanalyzer (Agilent, Santa Clara, CA). It was then quantified with NanoDrop ND-1000 Spectrophotometer (NanoDrop Technologies, Inc., Wilmington, DE).

### Adapter-ligation gDNA library amplification using real-time qPCR

7.5 μL PCR reactions were set up in a 384-well PCR plate (Bio-Rad, Hercules, CA) with 3.75 μL SYBR Green Supermix (Bio-Rad, Hercules, CA), 0.15 μL 50 μM i5 primer, 0.15 μL 50 μM i7 primer (Additional file 12: Table S2), and 3.45 μL (500, 250, 125 and 62.5 pg) adapter ligated DNA diluted in 1X Phosphate Buffer Saline adapter ligated DNA diluted in 1X Phosphate Buffer Saline. Cycling parameters were 72 °C for 3 min, 98 °C for 30 s, followed by 15 cycles of 10 s at 98 °C, 30 s at 65 °C and 1 min 45 s at 72 °C. The end RFU values were determined with CFX Manager software (Bio-Rad, Hercules, CA) for all libraries.

### Set-up for melting curve and pooling for adapter-ligated libraries and Nextera libraries

The PCRs were diluted with 2.5 μL water with the Biomek FX (Beckman Coulter, Indianapolis, IN). 7 μL of the diluted PCRs were transferred to low dead volume (LDV) plates (Labcyte, Sunnyvale, CA) compatible with the Labcyte Echo 550 (Labcyte, Sunnyvale, CA) leaving 3 μL of diluted PCR behind. The PCR plate was transferred to the CFX384 Touch Real-Time PCR Detection System (Bio-Rad, Hercules, CA) for MCA. The LDV plate was stored at − 20 °C until library pooling.

### Melting curve analysis adapter-ligated DNA and Nextera libraries

The PCR plates were incubated in the CFX384 Touch Real-Time PCR Detection System (Bio-Rad, Hercules, CA) for MCA, gradually ramping from 50 °C to 95 °C using increments of 0.5 °C, with 5 s at each temperature as fluorescence was monitored by CFX Manager software (Bio-Rad, Hercules, CA).

### Library pooling calculation adapter-ligated DNA and Nextera libraries

The transfer volume of each PCR reaction was calculated with the end RFU values determined with CFX Manager Software (Bio-Rad, Hercules, CA). To calculate the transfer volume of a given well, the minimum fluorescence volume was multiplied by the maximum allowable transfer volume and was then divided by the actual fluorescence value of that well.

### Library pooling

The LDV plate containing the diluted libraries was thawed to room temperature for library pooling. Each well was transferred to a 384-well PCR plate (Bio-Rad, Hercules, CA) with the Echo 550 (Labcyte, Sunnyvale, CA) using the transfer volumes calculated from the end RFU values. To avoid drip-back from the destination plate, the maximum volume transferred to each well in the 384-well PCR plate was 15 μL. The contents of each transfer well in the destination plate were then pooled together in a 1.7 mL tube (Eppendorf, Hamburg, Germany). Following pooling, the library was cleaned up with AMPure beads (Beckman Coulter, Indianapolis, IN) according to the manufacturer’s instructions. Library quality and size distribution was visualized on the Bioanalyzer (Agilent, Santa Clara, CA), and concentration was measured with Qubit dsDNA HS Assay Kit (Invitrogen, Carlsbad, CA).

### Illumina sequencing

The Nextera and adapter-ligated libraries were sequenced with the MiSeq Reagent Nano kit v2 (Illumina, San Diego, CA), following Illumina’s standard protocol. PhiX Control Library (v3) (Illumina, San Diego, CA) was included with the libraries as an internal sequencing control. The Nextera library and adapter-ligated libraries ran for two rounds of 150 or 100 cycles, respectively.

### Illumina sequencing analysis

Analysis of both sequencing runs was accomplished using embedded MiSeq Reporter (MSR) software (Illumina, San Diego, CA). The reads were aligned to the appropriate reference sequences with BWA-MEM [29]. Sequencing and alignment metrics were generated via MSR. For quality assessment of sequencing reads, BBTools Reformat [30] was used to generate quality value scores for individual libraries and the PhiX Control Library.

### FA-NGS software tool

The FA-NGS software tool was written in python using pandas, numpy, seaborn, and matplotlib.pyplot libraries. The code is divided into four classes: set-up, plates, analysis, and pooling calculator. Set-up is used to output a CSV file with Labcyte echo instrument instructions for how to set up the multiplexed PCR plates with single or dual indexed primers. The input is an excel file with plate layouts for source plate, reverse primer destinations, and forward primer destinations. The plates class is used to customize plate setups. This software tool can use 96 well plates, 384 well plates, quadrants of a 384 well plate, and is suitable for some customizable setups. Analysis is used to read and visualize end RFU measurements and MCA directly from the Bio-Rad CFX instrument output files. The pooling calculator reads the end RFU measurements output file to determine pooling quantities. The calculator outputs instructions for pooling with Labcyte Echo instruments as well as a visualization of transfer volumes per well. The FA-NGS software tool was written in python and is available for download. See the availability of data and materials section for additional information.

## Supplementary information

**Additional file 1: Figure S1.** Standard NGS and FA-NGS Workflow Comparison: Side-by-side comparison of the Standard NGS workflow (left) and modified FA-NGS workflow (right) highlights how FA-NGS can save time and hands on steps in preparing NGS libraries

**Additional file 2: Figure S2.** Input titration of Nextera library prepared plasmid monitored with FA: DNA diluted 4-fold starting at 100 pg per Nextera tagmentation reaction was amplified with SYBR green and monitored by continuous fluorescence (A), and melting curve analysis (B)

**Additional file 3: Figure S3.** MCA maxima correlates with input titration: Amount of input DNA is correlated with the local maxima of the MCA determined derivative RFU of the input titrations of both Nextera (A) and AL (B) library prepared samples, with R2 equal to 0.807 and 0.842 respectively

**Additional file 4: Figure S4.** Distributions of end RFU and transfer volumes of AL libraries: End RFU values (A) and transfer volumes (B) of AL libraries generated with 500 (blue), 250 (red), 125 (yellow), and 62.5 (green) pg of input AL DNA are represented in a histogram. For adjacent histograms, * represents distributions with p-value < 0.001

**Additional file 5: Figure S5.** Melting curve analysis of Nextera prepared plasmids: The melting curve plot (temperature vs. negative derivative of fluorescence (−dF/dT)) of every well from the Nextera library is plotted. From left to right, the plasmids tested are pXMJ19, pskb3-CopR1598, pGEN-292, pms6126

**Additional file 6: Figure S6.** Melting curve analysis of AL prepared gDNA: The melting curve plot (temperature vs. negative derivative of fluorescence (−dF/dT)) of every well from the AL-gDNA library is plotted. From quadrant 1–4, the input concentrations are 500 pg, 250 pg, 125 pg, 62.5 pg

**Additional file 7: Figure S7.** Comparison of percent reads between Nextera and AL libraries shows similarities in output from two distinct NGS workflows: The distributions of Nextera (blue) and AL (red) libraries of percent reads are overlaid to highlight the similarities (p-value = 1) of sequencing output from these methods. The range of percent reads for the Nextera library (blue) was 0.39–1.95, with a mean of 1.04 and a standard deviation of 0.43. The range of percent reads for the AL library (red) was 0.25–2.89, with a mean of 1.04 and a standard deviation of 0.5

**Additional file 8: Figure S8.** Percent difference from sequence pooling of Nextera and AL libraries: The frequency of percent differences from the expected percent reads per sample (1.04) is represented as a histogram for the Nextera library (A), AL library (B)

**Additional file 9: Figure S9.** Sequencing quality scores of Nextera and AL libraries: The percentage of bases with ≥ Q30 quality score for PhiX Control Library and for Nextera and AL libraries demonstrates sequencing quality for FA-NGS libraries

**Additional file 10: Figure S10.** Continuous fluorescence measurements of qPCR: RFU values per cycle number are plotted for 96 plasmid libraries (A) and 96 gDNA libraries (B)

**Additional file 11: Table S1.** Sequencing analysis for Nextera library: alignment analysis was performed using embedded MiSeq Reporter software

**Additional file 12: Table S2.** Primers for amplification of AL library

## Data Availability

The FA-NGS software tool was written in python and is available for download at: https://github.com/AgileBioFoundry/FA-NGS. All plasmids used are available through the public instance of the ABF registry: (https://public-registry.agilebiofoundry.org/folders/2) [18]. DNA sequencing was deposited in the Sequence Read Archive (SRA) database of the National Center for Biotechnology Information (NCBI) with Bioproject PRJNA599152.

## Abbreviations

FA-NGS: Fluorescent amplification for next generation sequencing
GDNA: Genomic DNA
MCA: Melting curve analysis
MSR: MiSeq Reporter
NGS: Next generation sequencing
QC: Quality control
QPCR: Quantitative PCR
RFU: Relative fluorescence unit
