# Supplementary figures and images for: Fluorescent amplification for next generation sequencing (FA-NGS) library preparation
Source: BMC Genomics. 2020 Jan 28;21:85. doi: 10.1186/s12864-020-6481-8 (PMC6988211; doi:10.1186/s12864-020-6481-8)

## Standard NGS workflow

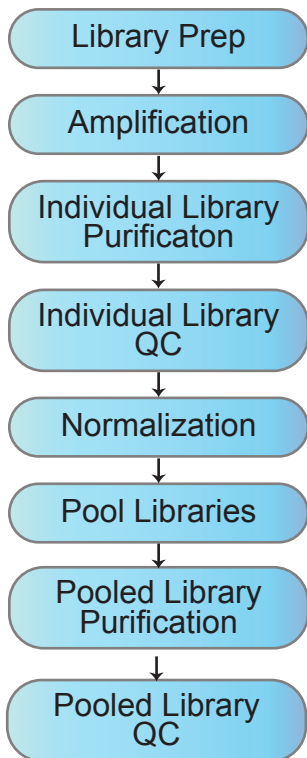

## FA-NGS workflow

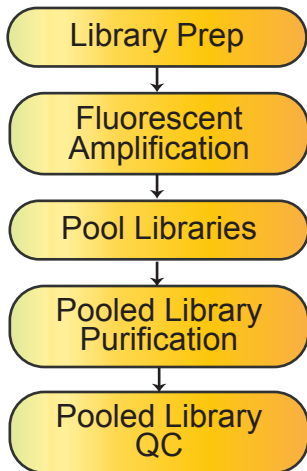

Supplementary Figure 1

Supplement: Supplementary file 1 — Additional file 1: Figure S1. Standard NGS and FA-NGS Workflow Comparison: Side-by-side comparison of the Standard NGS workflow (left) and modified FA-NGS workflow (right) highlights how FA-NGS can save time and hands on steps in preparing NGS libraries [file 12864_2020_6481_MOESM1_ESM.pdf]

(A)

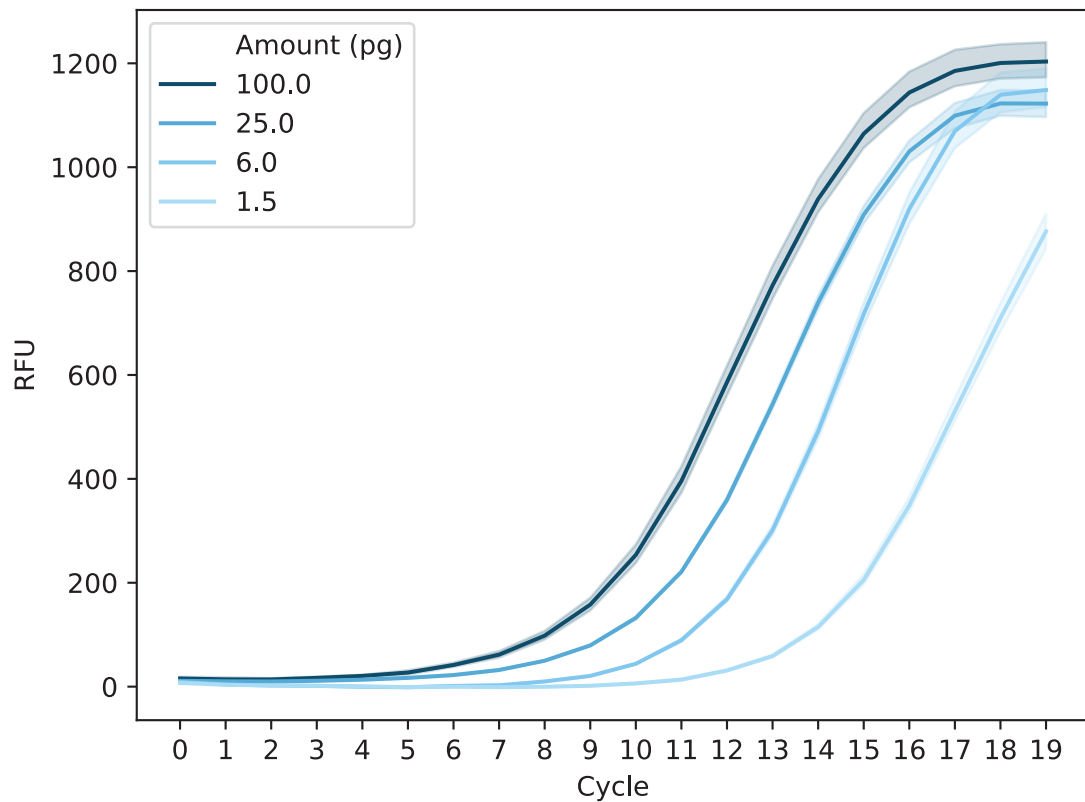

(B)

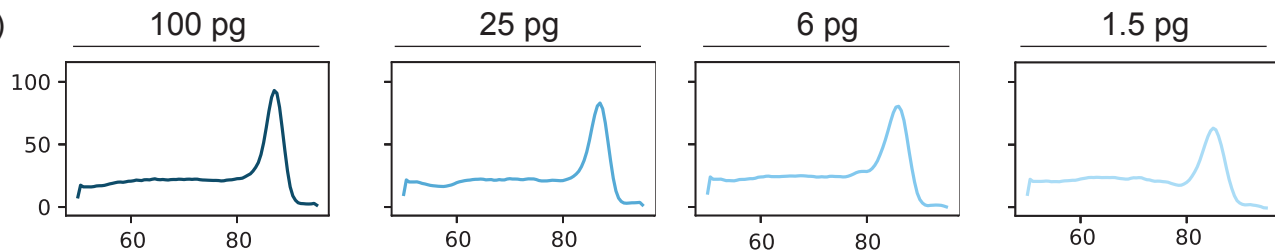

Supplementary Figure 2

Supplement: Supplementary file 2 — Additional file 2: Figure S2. Input titration of Nextera library prepared plasmid monitored with FA: DNA diluted 4-fold starting at 100 pg per Nextera tagmentation reaction was amplified with SYBR green and monitored by continuous fluorescence (A), and melting curve analysis (B) [file 12864_2020_6481_MOESM2_ESM.pdf]

(A)

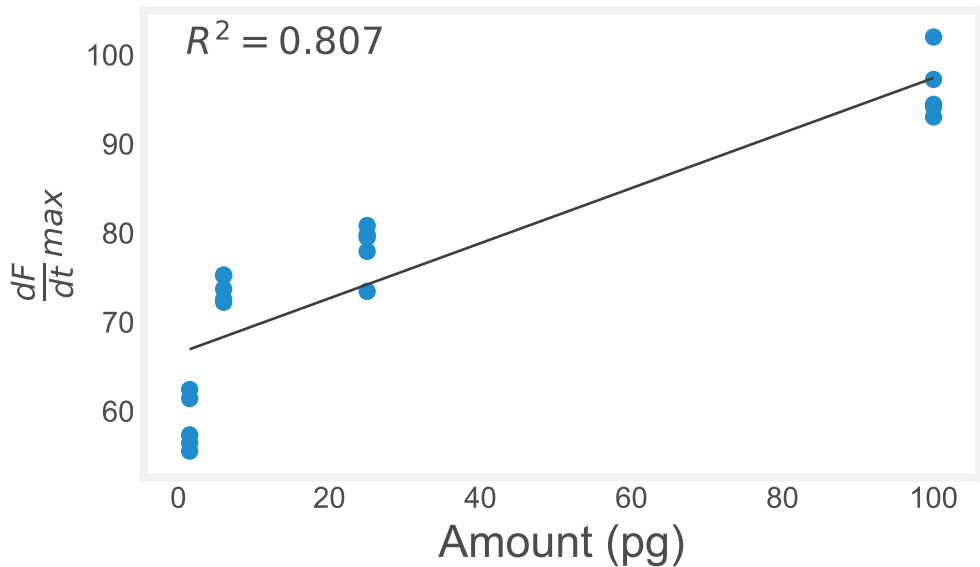

(B)

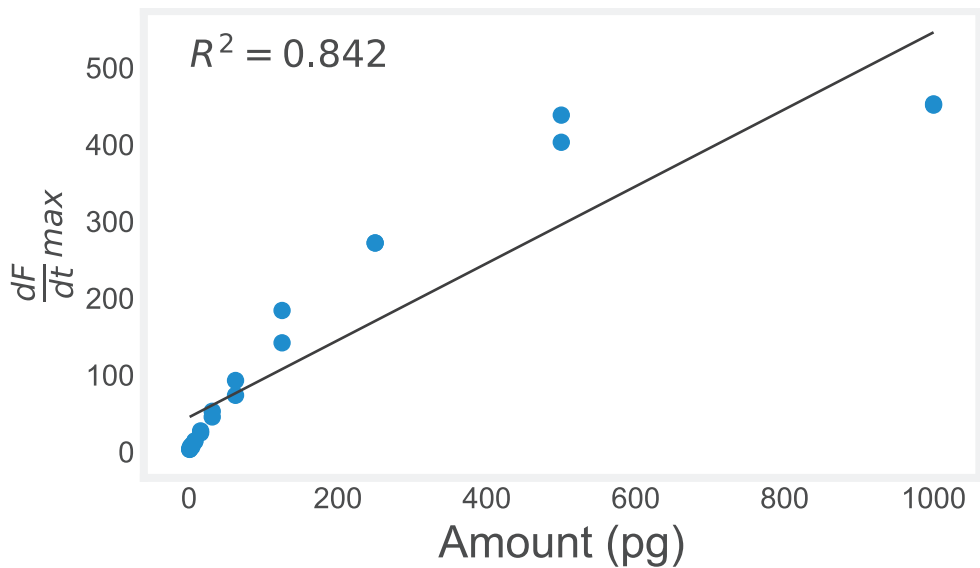

Supplement: Supplementary file 3 — Additional file 3: Figure S3. MCA maxima correlates with input titration: Amount of input DNA is correlated with the local maxima of the MCA determined derivative RFU of the input titrations of both Nextera (A) and AL (B) library prepared samples, with R2 equal to 0.807 and 0.842 respectively [file 12864_2020_6481_MOESM3_ESM.pdf]

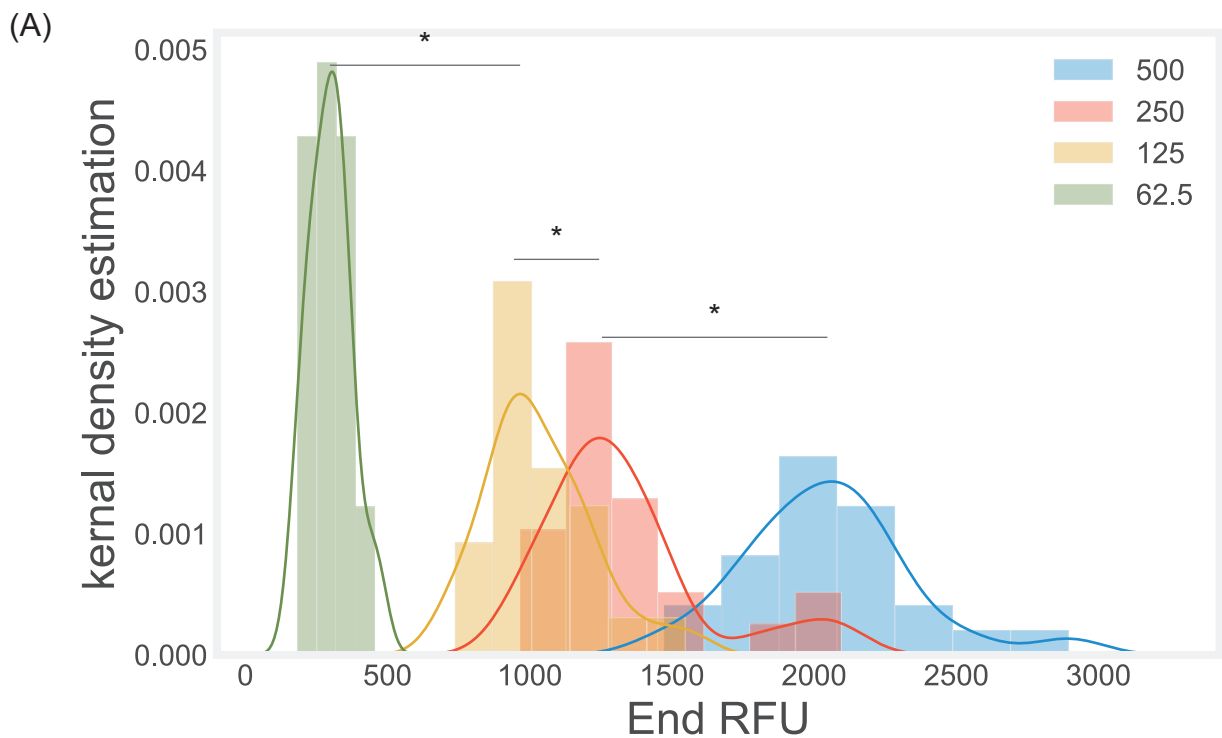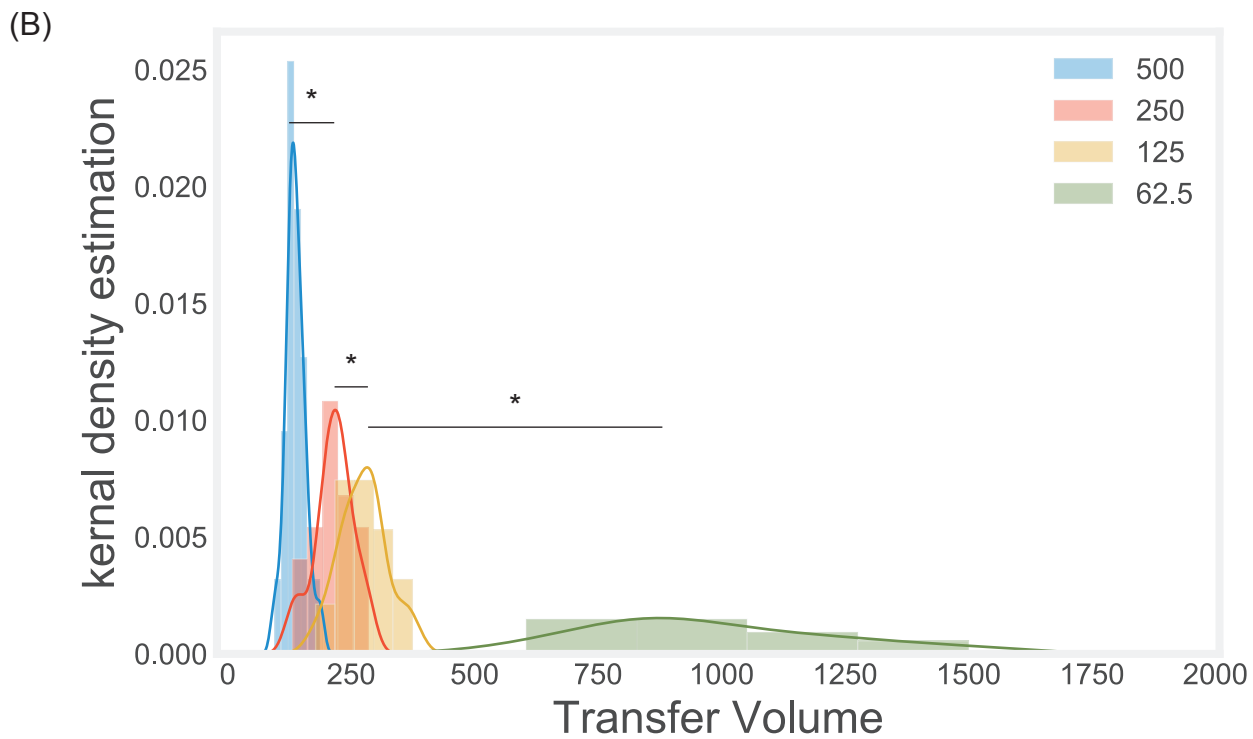

Supplementary Figure 4

Supplement: Supplementary file 4 — Additional file 4: Figure S4. Distributions of end RFU and transfer volumes of AL libraries: End RFU values (A) and transfer volumes (B) of AL libraries generated with 500 (blue), 250 (red), 125 (yellow), and 62.5 (green) pg of input AL DNA are represented in a histogram. For adjacent histograms, * represents distributions with p-value < 0.001 [file 12864_2020_6481_MOESM4_ESM.pdf]

$-\text{dF}/\text{dT}$

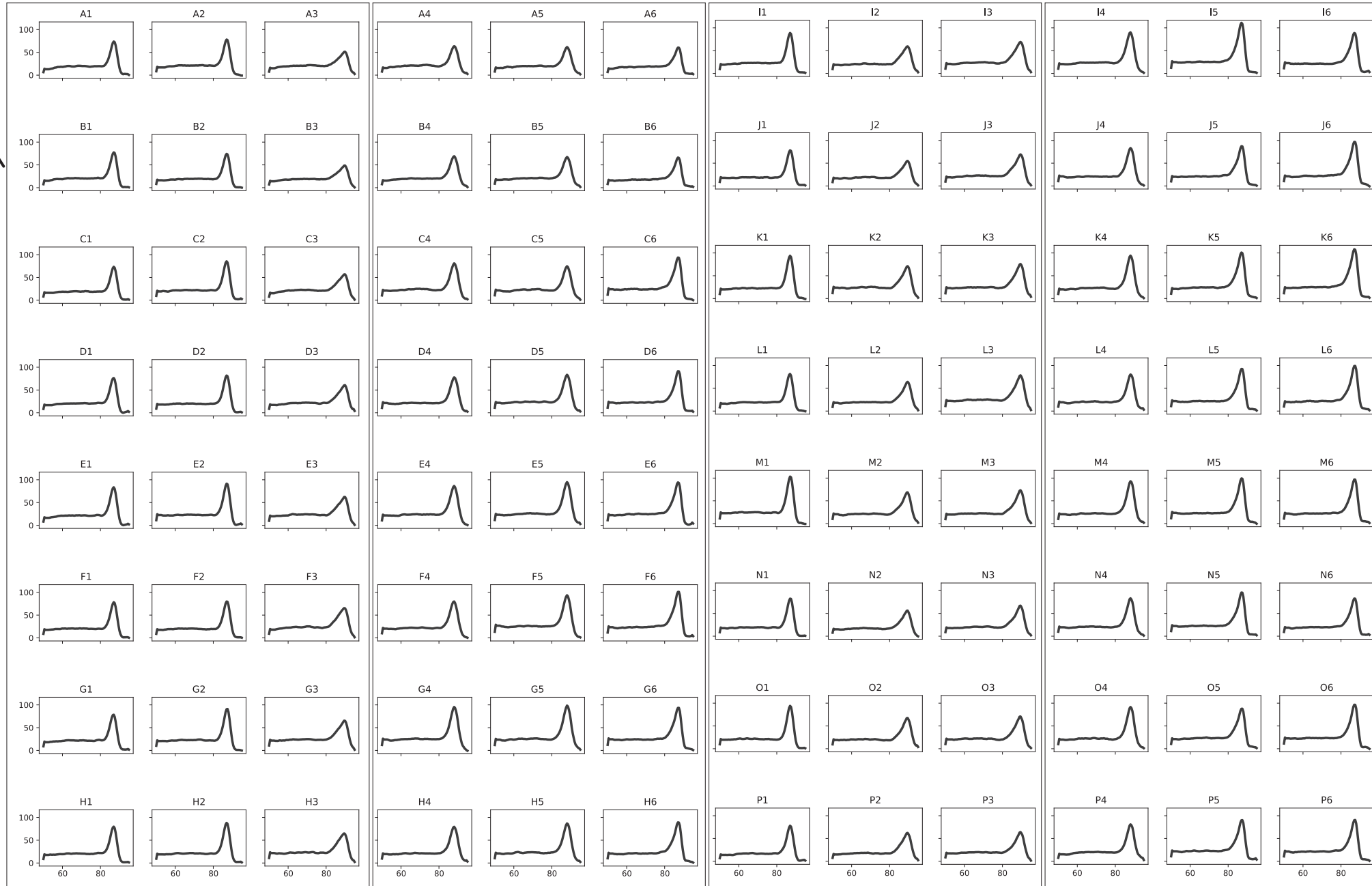

Temperature (°C)

Supplement: Supplementary file 5 — Additional file 5: Figure S5. Melting curve analysis of Nextera prepared plasmids: The melting curve plot (temperature vs. negative derivative of fluorescence (−dF/dT)) of every well from the Nextera library is plotted. From left to right, the plasmids tested are pXMJ19, pskb3-CopR1598, pGEN-292, pms6126 [file 12864_2020_6481_MOESM5_ESM.pdf]

$-dF/dT$

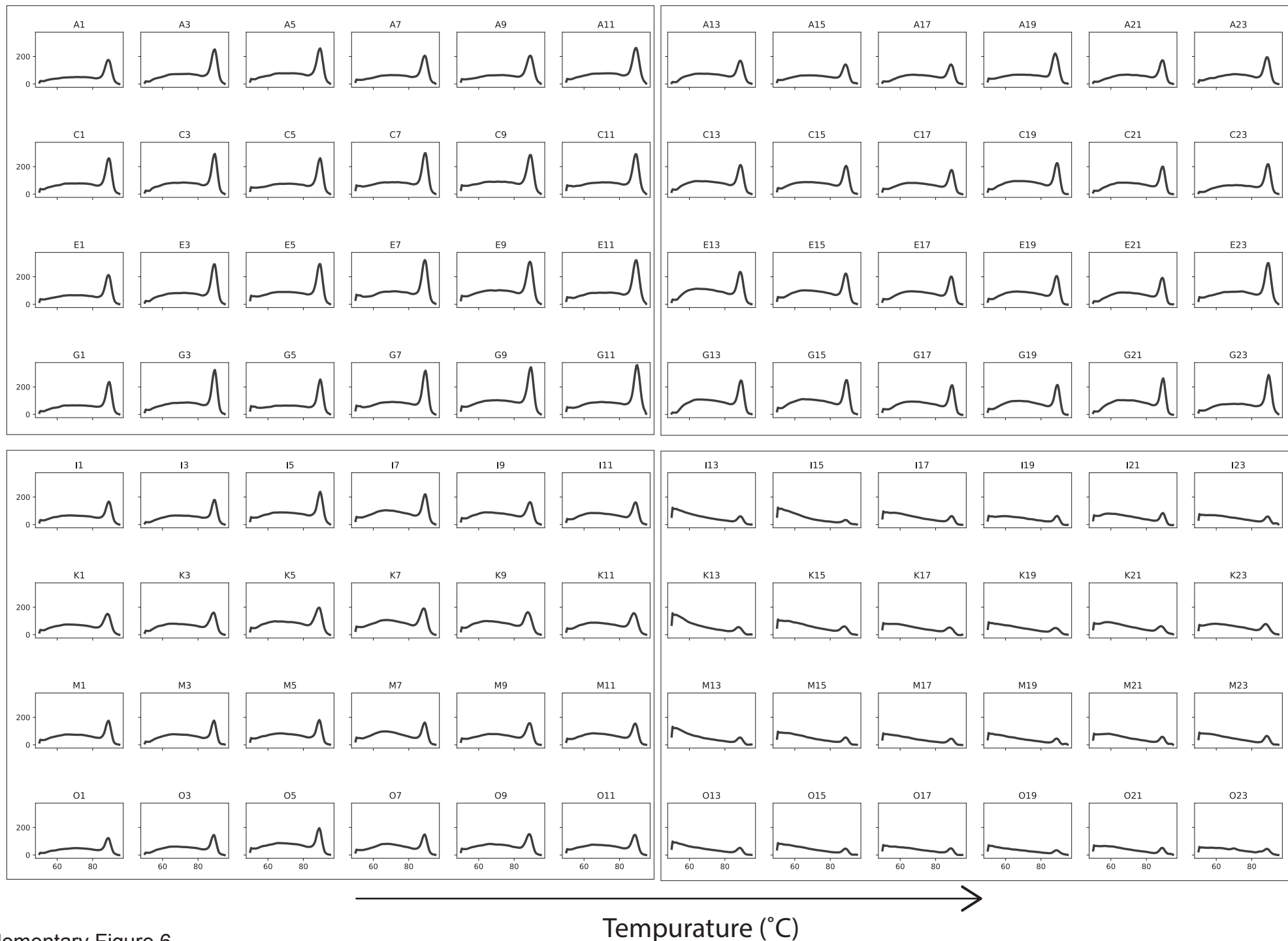

Supplementary Figure 6

Supplement: Supplementary file 6 — Additional file 6: Figure S6. Melting curve analysis of AL prepared gDNA: The melting curve plot (temperature vs. negative derivative of fluorescence (−dF/dT)) of every well from the AL-gDNA library is plotted. From quadrant 1–4, the input concentrations are 500 pg, 250 pg, 125 pg, 62.5 pg [file 12864_2020_6481_MOESM6_ESM.pdf]

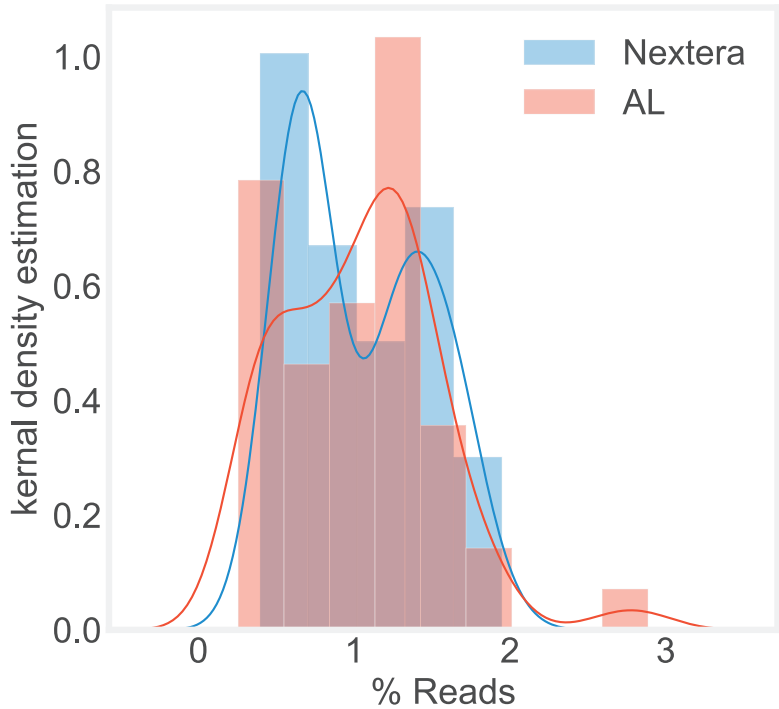

Supplementary Figure 7

Supplement: Supplementary file 7 — Additional file 7: Figure S7. Comparison of percent reads between Nextera and AL libraries shows similarities in output from two distinct NGS workflows: The distributions of Nextera (blue) and AL (red) libraries of percent reads are overlaid to highlight the similarities (p-value = 1) of sequencing output from these methods. The range of percent reads for the Nextera library (blue) was 0.39–1.95, with a mean of 1.04 and a standard deviation of 0.43. The range of percent reads for the AL library (red) was 0.25–2.89, with a mean of 1.04 and a standard deviation of 0.5 [file 12864_2020_6481_MOESM7_ESM.pdf]

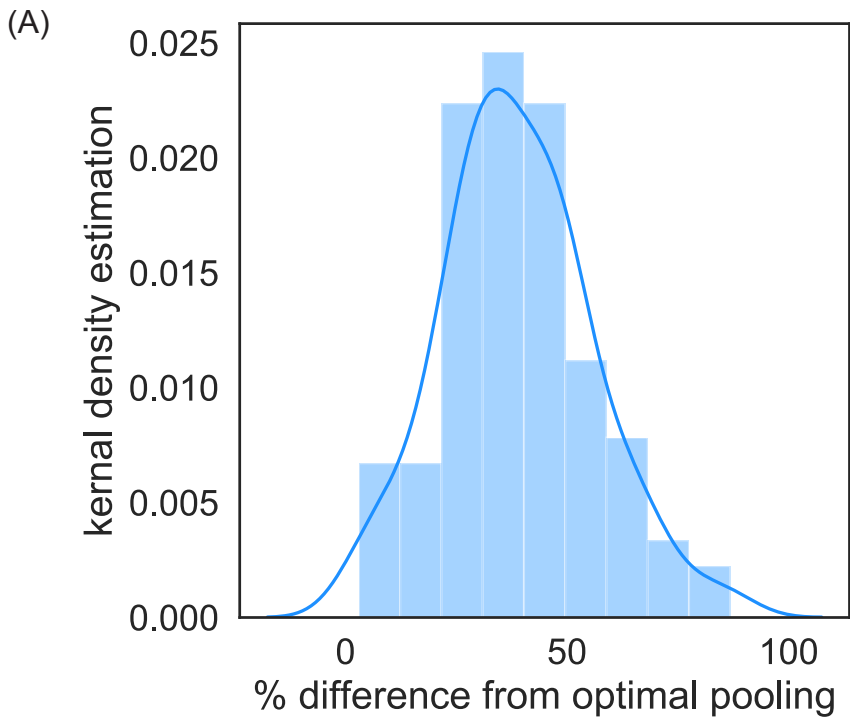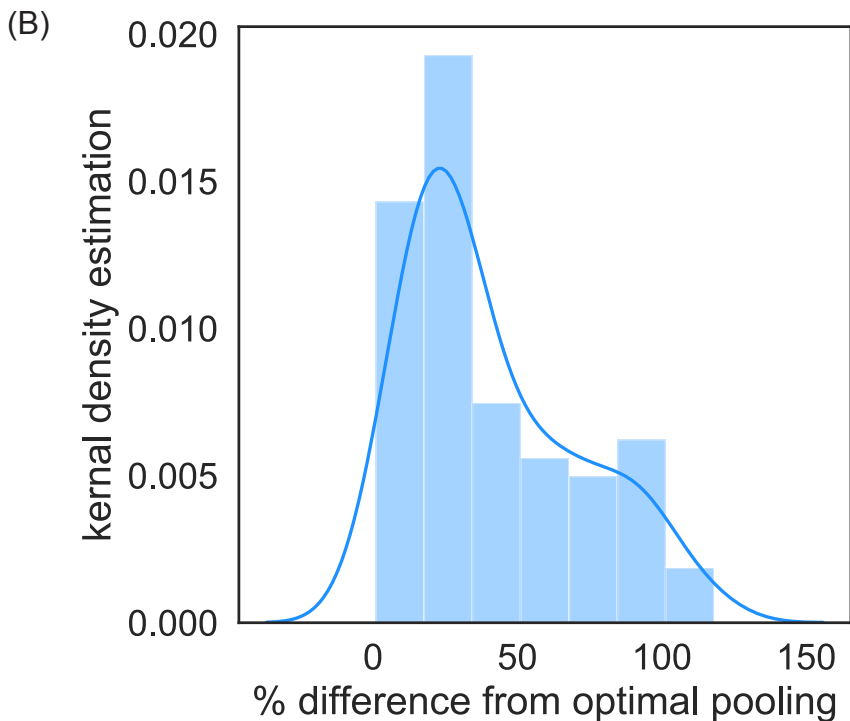

Supplementary Figure 8

Supplement: Supplementary file 8 — Additional file 8: Figure S8. Percent difference from sequence pooling of Nextera and AL libraries: The frequency of percent differences from the expected percent reads per sample (1.04) is represented as a histogram for the Nextera library (A), AL library (B) [file 12864_2020_6481_MOESM8_ESM.pdf]

(A)

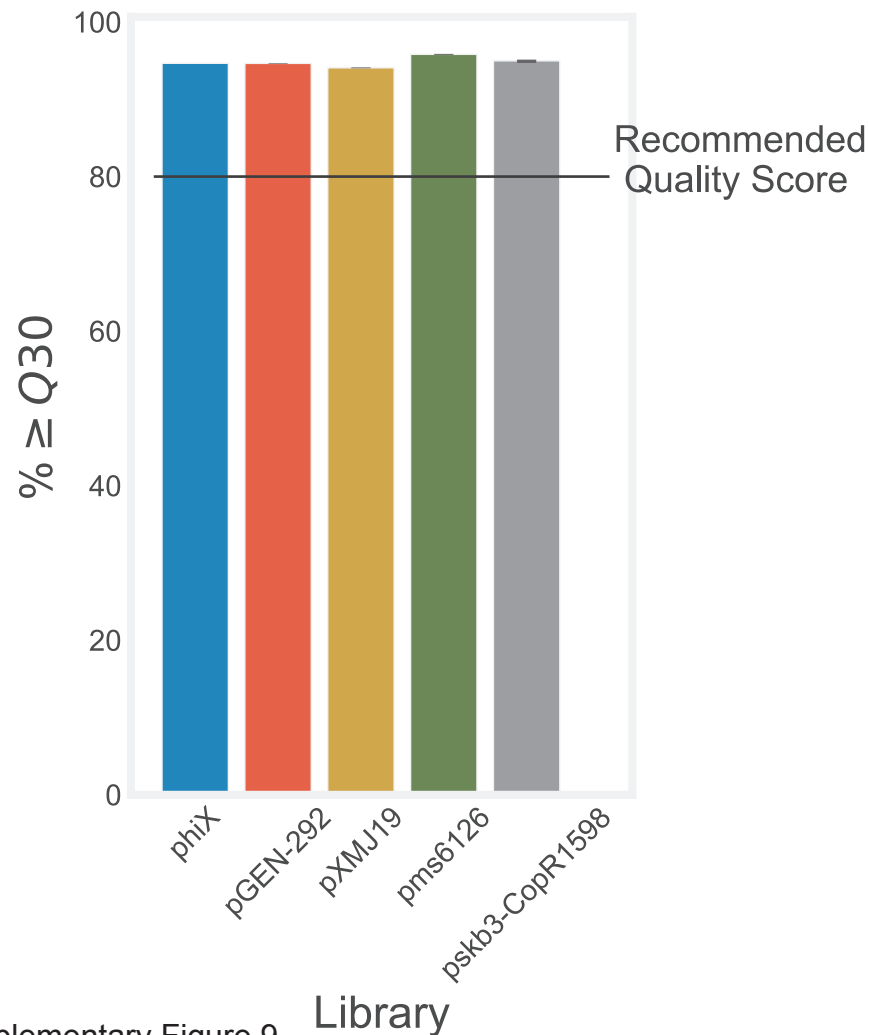

(B)

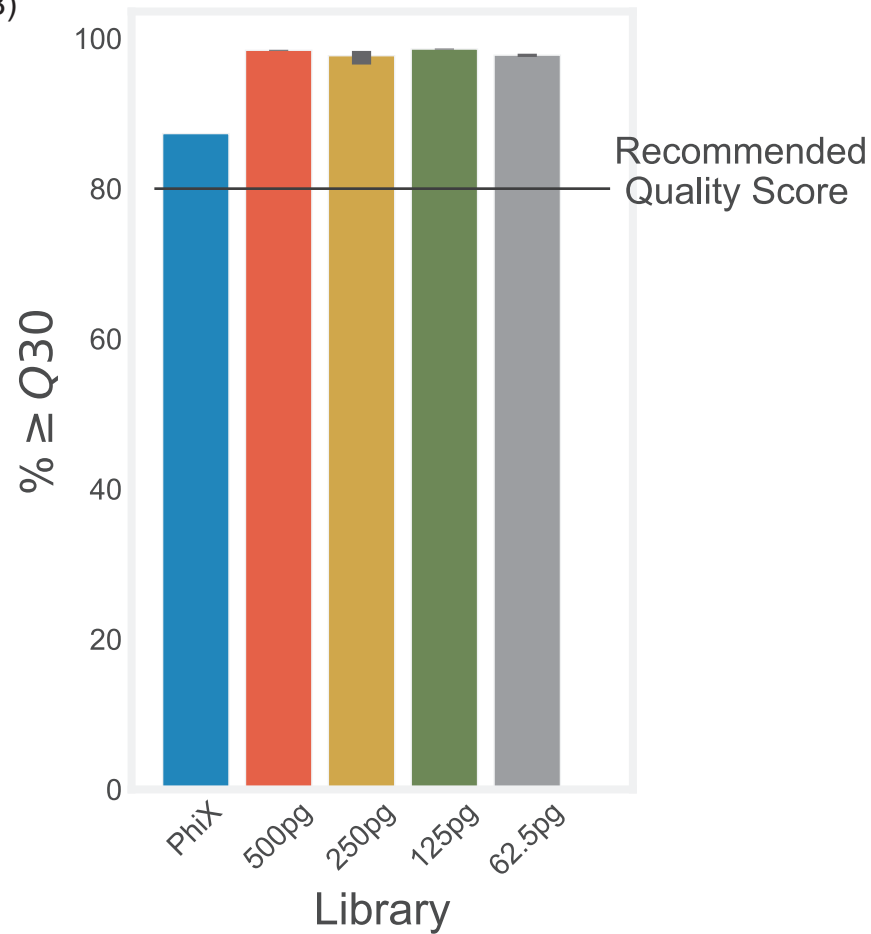

Supplement: Supplementary file 9 — Additional file 9: Figure S9. Sequencing quality scores of Nextera and AL libraries: The percentage of bases with ≥ Q30 quality score for PhiX Control Library and for Nextera and AL libraries demonstrates sequencing quality for FA-NGS libraries [file 12864_2020_6481_MOESM9_ESM.pdf]

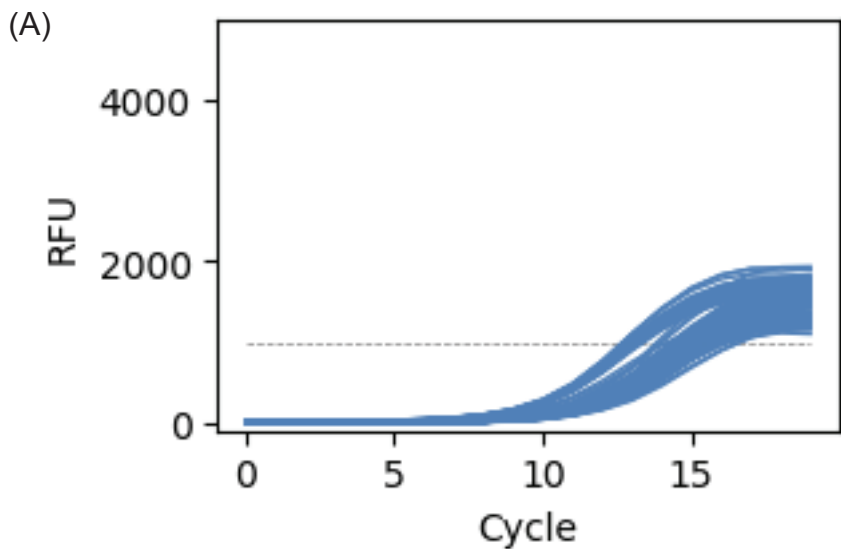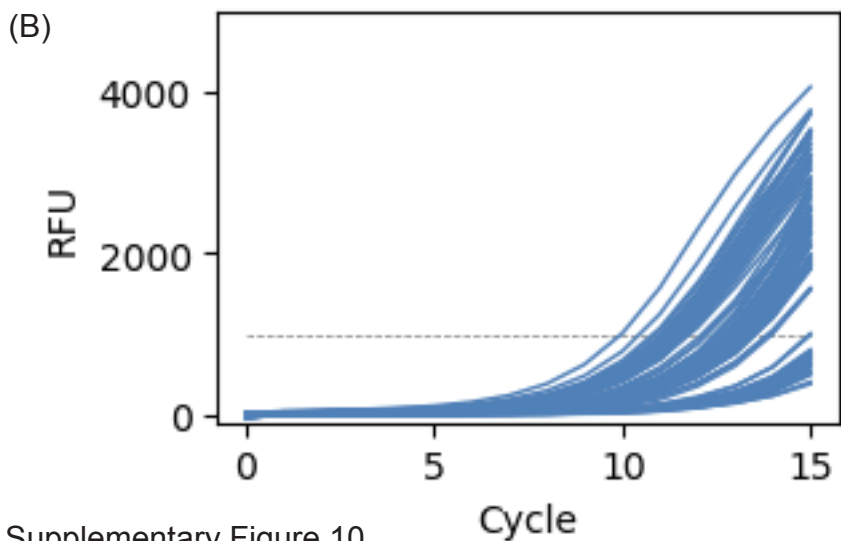

Supplementary Figure 10

Supplement: Supplementary file 10 — Additional file 10: Figure S10. Continuous fluorescence measurements of qPCR: RFU values per cycle number are plotted for 96 plasmid libraries (A) and 96 gDNA libraries (B) [file 12864_2020_6481_MOESM10_ESM.pdf]
